# Benefits for Voice Learning Caused by Concurrent Faces Develop over Time

**DOI:** 10.1371/journal.pone.0143151

**Published:** 2015-11-20

**Authors:** Romi Zäske, Constanze Mühl, Stefan R. Schweinberger

**Affiliations:** 1 Department for General Psychology and Cognitive Neuroscience, Institute of Psychology, Friedrich Schiller University of Jena, Jena, Germany; 2 School of Psychology, Bangor University, Bangor, Gwynedd, Wales, United Kingdom; Harvard Medical School/Massachusetts General Hospital, UNITED STATES

## Abstract

Recognition of personally familiar voices benefits from the concurrent presentation of the corresponding speakers’ faces. This effect of audiovisual integration is most pronounced for voices combined with dynamic articulating faces. However, it is unclear if *learning* unfamiliar voices also benefits from audiovisual face-voice integration or, alternatively, is hampered by attentional capture of faces, i.e., “face-overshadowing”. In six study-test cycles we compared the recognition of newly-learned voices following unimodal voice learning vs. bimodal face-voice learning with either static (Exp. 1) or dynamic articulating faces (Exp. 2). Voice recognition accuracies significantly increased for bimodal learning across study-test cycles while remaining stable for unimodal learning, as reflected in numerical costs of bimodal relative to unimodal voice learning in the first two study-test cycles and benefits in the last two cycles. This was independent of whether faces were static images (Exp. 1) or dynamic videos (Exp. 2). In both experiments, slower reaction times to voices previously studied with faces compared to voices only may result from visual search for faces during memory retrieval. A general decrease of reaction times across study-test cycles suggests facilitated recognition with more speaker repetitions. Overall, our data suggest two simultaneous and opposing mechanisms during bimodal face-voice learning: while attentional capture of faces may initially impede voice learning, audiovisual integration may facilitate it thereafter.

## Introduction

Facilitated processing of audiovisual relative to unimodal stimuli is commonly attributed to integration of congruent bimodal information. This has not only been shown for linguistic properties of speech (e.g. [1]), but also for non-linguistic socially relevant cues in voices and faces (reviewed in [2]). For instance, recognizing familiar voices is facilitated by the presence of the corresponding faces compared to voice-only recognition [3], whereas familiar voice recognition in that study was hampered by the presence of non-corresponding faces when these were shown as synchronized videos. The pattern of larger benefits and costs for videos showing faces articulating in time-synchrony, compared with static faces, suggested a mechanism of face-voice integration. Since voices and faces convey identity-specific physical characteristics of a speaker’s articulatory movements, concurrent presentation of faces and voices may aid familiar speaker recognition by providing redundant dynamic cues to speaker identity. It is assumed by current models of person perception [4, 5] that these cues can trigger multimodal identity representations for familiar speakers.

However, audiovisual integration of dynamic face-voice information has also been reported in unfamiliar speaker perception [6]. Specifically, that study reported above-chance accuracy for a voice-face identity matching task, but only when voices had to be matched to videos of silently articulating faces, rather than static face images. While the above studies suggest a role of audiovisual integration for familiar voice recognition and unfamiliar face-voice matching, it is less clear if unfamiliar voice learning is also facilitated, unaffected, or even hampered by the concurrent presentation of (dynamic) faces relative to voices only. From the above research one would predict that faces support voice learning by facilitating the acquisition of multimodal speaker representations. Unlike unimodal voice representations, multimodal speaker representations may then assist subsequent voice recognition by additionally storing facial cues to speaker identity. If so, this effect should even be more pronounced for dynamic than for static study faces, to the extent that the former share redundant dynamic information with voices and may thus be preferentially integrated.

In fact, the few studies that have compared voice recognition following unimodal voice learning and bimodal face-voice learning produced mixed results. For instance, there is evidence that, compared to voice-only learning, concurrent dynamic faces may either facilitate [7] or impair [8] subsequent voice recognition. Similarly, static faces presented along with study voices have been reported to either have no effect [9, 10] or impair subsequent voice-only recognition [11, 12]. Impaired voice recognition after audiovisual learning has been discussed to result from face-overshadowing [8, 12], such that faces capture attention to a larger extent than do voices thereby interfering with voice encoding during study. As before, it could be expected that this interference effect is increased for dynamic faces since the onset of visual motion usually captures attention more readily than do static stimuli [13]. However, to the best of our knowledge there are currently no studies comparing the effects of voice learning with static vs. dynamic faces on subsequent unimodal voice recognition.

Taken together, while current models of person perception predict benefits of audiovisual integration during voice learning for subsequent voice recognition [5], an alternative prediction is that faces interfere with voice learning thereby impeding subsequent voice recognition [8, 12]. To shed more light on these opposing hypotheses, we conducted two experiments that compare voice recognition with an old/new task after unimodal voice-only (V) learning and bimodal face-voice (FV) learning with either static faces (Exp. 1) or dynamically articulating faces (Exp. 2). In both experiments, bimodal learning effects (FV minus V) were assessed in six consecutive study-test cycles. This was in order to explore the progression of voice recognition and the nature of bimodal learning effects across several study-test repetitions.

## Methods

### Participants

In Experiment 1, 16 participants (aged between 19 and 28 years; *M* = 21.8 yrs; *SD* = 2.5 yrs; 8 female; all right-handed) and in Experiment 2, 16 new participants (aged between 19 and 29 years; *M* = 22.7 yrs; *SD* = 3.0 yrs; 8 female; all right-handed) contributed data. All participants were native German speakers and none reported hearing problems or prior familiarity with any of the voices. Data from 7 further participants were excluded from the analyses due to technical problems (*N* = 1 for Exp. 1 and 2 respectively) or prior familiarity with speakers (*N* = 2 in Exp. 1; *N* = 3 in Exp. 2). All participants gave written informed consent and received course credit or €5. The study was conducted in accordance with the Declaration of Helsinki and approved by the Ethics Committee of the University of Jena.

### Stimuli

Stimuli were audio and video recordings as well as photographs from 24 young speakers (aged between 18 and 22 years; *M* = 20.7 yrs; *SD* = 1.3 yrs; 12 female) recruited among students of the University of Jena. Speakers received €5 for participation and gave their written informed consent to the use of their voices and faces as stimuli in our experiments. From each speaker we simultaneously recorded face videos and voices by means of a Sony DCR-DVD403E camcorder and a Sennheiser MD 421-II Microphone, respectively. The microphone was connected to a CEntrance MicPort-Pro pre-amplifier and a SoundMax HD Audio soundcard. To standardize videos, speakers sat on a chair in front of a black background and were illuminated by a three-point lighting system. All speakers were instructed to take off glasses, jewelry and make-up and, if necessary, to shave before the session. Furthermore, speakers were asked to look directly into the camera with a neutral facial expression and to close their mouth before each utterance. As part of a bigger protocol each speaker uttered a set of three German sentences: “Du bist doch, was Du denkst” (“You are indeed what you think”); “Keine Antwort ist auch eine Antwort” (“No answer is an answer as well”); “Dichter und Denker dachten dasselbe” (“Poets and thinkers thought the same”). To standardize articulation speakers were asked to produce the sentences in similar style and timing, and in emotionally neutral intonation, as exemplified in a prerecorded model speaker (third author). Each utterance was recorded 4–5 times to choose the best take (no artifacts, clear articulation and neutral expression). Note that for this reason, we selected audio and video samples independently such that the audio and video of a given speaker and sentence did not necessarily belong to the same take.

Using Adobe Audition 1.5 and Adobe Premiere CS4 respectively, voice samples (16-bit resolution, 44.1 kHz sampling rate) and videos (25fps) were cut to contain one sentence per file beginning exactly at plosive onset. Following RMS-normalization of audio recordings using PRAAT [14], all voice samples were time-standardized with respect to overall duration and all word onsets within each of the three sentences. This was also done with videos such that video and audio tracks could be recombined in perfect time-synchrony [15] to create time-standardized bimodal learning stimuli for the face-voice (FV) condition of Exp 2. Time-standardization was based on mean overall duration and word onsets across both audios and videos of all 24 speakers. This was done for each of the three sentences separately. Mean timings were then rounded to match the frame rate of the videos such that the overall duration and onset of every word were even multiples of the frame duration (40ms). Time-standardization was achieved by applying the stretching function both to videos and audios, with original voice pitch being preserved using Adobe Premiere CS4 and Adobe Audition 1.5 respectively. After combining faces and voices of corresponding speakers into a video, an elliptic mask was superimposed and centered on the speakers’ faces to eliminate clothing information as a potential cue to speaker identity. To create bimodal learning stimuli for Exp. 1 the first frame of each video was rendered into a bmp-file showing a static face which was presented along with the voice. The same audio tracks that had been recombined with the videos and photos were used as unimodal learning stimuli in voice-only (V) conditions and as test stimuli in the voice recognition tests of both experiments. Speakers were set into four random sets of six speakers (three female) to serve as study and test speakers. As practice trials we used stimuli of four additional speakers (two female) not presented in the main experiment.

### Procedure

Participants were tested individually in a dimly-lit and sound attenuated chamber. Instructions were presented in writing on a computer screen and the experimenter did not talk to participants during the experiment to avoid interference from the experimenter’s voice. Audio stimuli were presented via Sennheiser HD212Pro headphones at a constant and comfortable hearing level and visual stimuli were presented via a computer screen at a standardized viewing distance of ~88 cm as ensured by a chin-rest. Faces (static and dynamic) subtended a visual angle of ~6.4° x 5.5° (height x width).

Both experiments consisted of two blocks with one block for each modality condition, i.e. the unimodal voice-only (V) and bimodal face-voice (FV) condition. Blocks comprised six study-test cycles with one study phase and a subsequent test phase per cycle. Across the six cycles of a respective block, all study phases contained identical stimuli presented in random order. The same was true for all test phases of each block.

**Fig 1:**
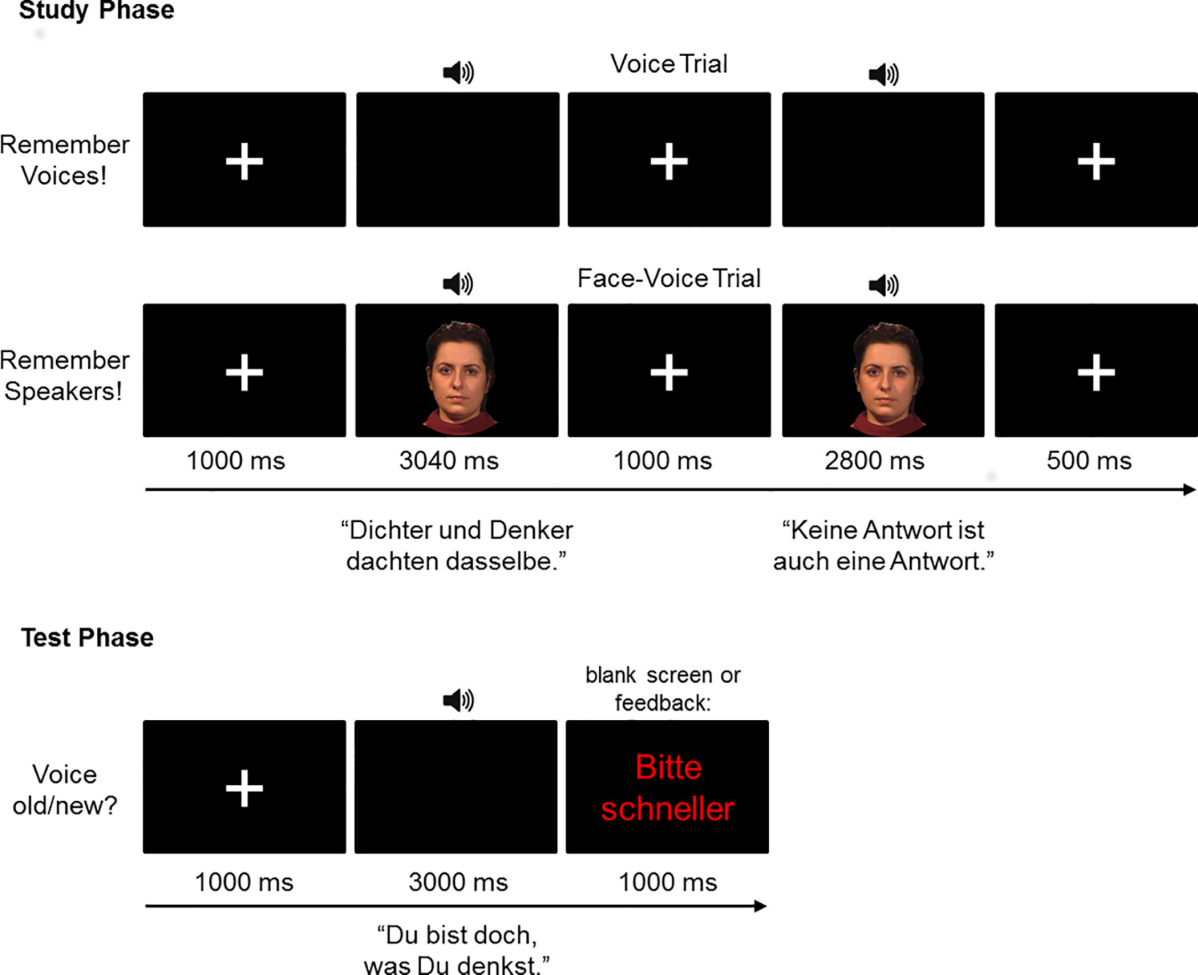
Top: Trial procedure for the study phases depicted for the voice-only block (V) and the face-voice block (FV). Bottom: Trial procedure in the test phases following V and FV learning. Note that face stimuli were static pictures in Exp. 1 and dynamic videos in Exp. 2. The individual depicted in this figure has given written informed consent (as outlined in PLOS consent form) to publish this photograph.

In each study phase of a given block participants were presented with a set of six speakers (three female) once, in random order. On each of these six study trials a speaker uttered two consecutive sentences („Dichter und Denker dachten dasselbe”and „Keine Antwort ist auch eine Antwort“) with order of sentences being constant across trials (cf. Fig 1, The individual depicted in Fig 1 has given written informed consent (as outlined in PLOS consent form) to publish this photograph.). Participants were instructed to remember the voices (V) or speakers (FV) for a subsequent test. Depending on modality block study voices were presented on their own (V) or with concurrent faces (FV). Different sets of study speakers were used in the two modality blocks. In total, each block comprised 72 study trials (6 study voices x 6 study-test cycles).

In the unimodal condition (V) study trials started with a white fixation cross (1000 ms) on a black background which remained on the screen during the subsequent presentation of the first voice sample (3040 ms), silence (1000 ms) and the second voice sample (2800 ms). Trials terminated with a blank screen (500 ms). Trial procedures were identical in both modality conditions with one exception: during voice presentation in the bimodal condition (FV), the fixation cross was replaced with the photo of a static face (Exp. 1) or the video of a dynamic face articulating in time-synchrony with the voice (Exp. 2). No responses were required on study trials. Total trial duration was thus 8340 ms. There were 72 study trials (6 study voices x 6 study-test cycles) in each modality block.

In each test phase of a given block participants made speeded old/new responses to 12 voices presented in random order, i.e. participants were to indicate as fast and as accurately as possible whether or not a given test voice had been presented in the previous study block (cf. Fig 1). All voices uttered the same sentence not used in the study phase („Du bist doch, was du denkst“). Of the 12 test voices, six (old) voices had been presented during the study phase and six (novel) voices were novel. Different sets of „novel”test speakers were used in the two modality blocks. No faces were presented at test.

Test trials started with a white central fixation cross (1000 ms) followed by the test voice sample (2280 ms). Using “D” and “L” keys on a computer keyboard (German layout), participants were instructed to classify test voices as studied or novel as fast and as accurately as possible. For responses slower than 3000 ms after voice onset a feedback (“Faster please!”) appeared on the screen (1000 ms). Responses given within this time window were followed by a blank screen (1000 ms). Total trial duration was thus 5000 ms. There were 144 test trials (12 study voices x 6 study-test cycles) in each modality block.

Each modality block was preceded by one study-test cycle for practice, containing four speakers not used in the main experiment. Order of modality blocks was counterbalanced across participants, as was the assignment of responses to response keys and of speaker sets to modality conditions (V and FV) as well as voice novelty conditions (studied vs. novel voices). Design and speaker identities were identical in both experiments. Overall, each experiment took ~40 min.

## Results

We analyzed voice recognition performance at test after excluding a small proportion of omission errors and responses faster than 200 ms from voice onset (1.04% and 1.56% of responses in Exp. 1 and 2 respectively). To assess the bimodal learning effect across study-test cycles with a substantial number of trials, we collapsed data across two adjacent cycles to obtain 3 cycle pairs. We then analyzed d-prime (d’), response criterion (C), accuracies and correct reaction times (RTs) using mixed-measures ANOVAs. For the d’ analysis, hit rates and false alarm rates equal to zero or one were adjusted according to MacMillan and Kaplan [16]. Where appropriate, Epsilon corrections for heterogeneity of covariances [17] were performed throughout.

**Fig 2:**
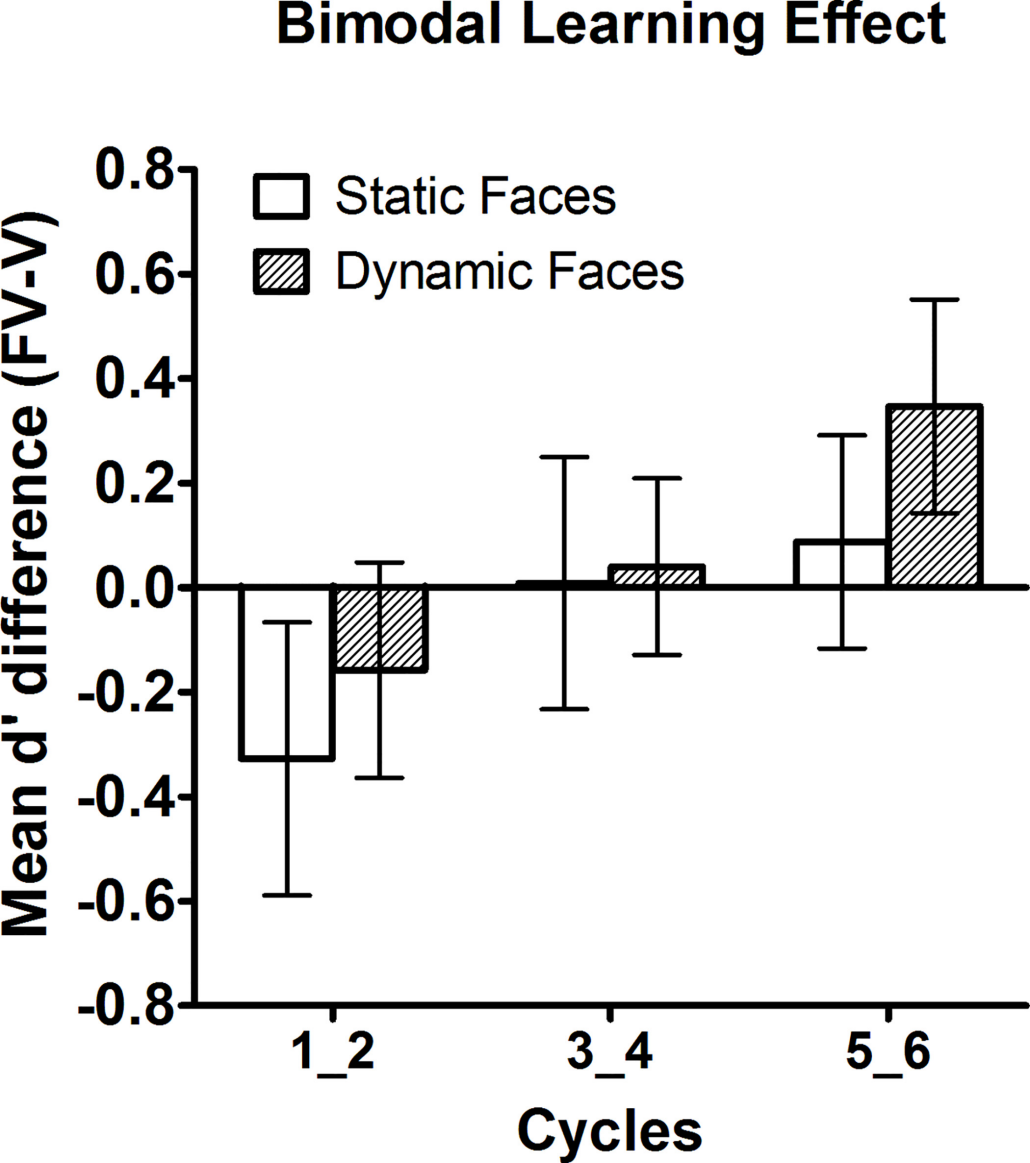
Bimodal learning effect depicted as mean d’- differences between face-voice (FV) and voice-only (V) modality conditions for pairs of consecutive study-test cycles in Exp. 1 (static faces) and Exp. 2 (dynamic faces). Note that increasing benefits of bimodal learning from cycle pairs 1_2 towards 5_6 were independent of face animation mode (static vs. dynamic). Error bars are standard errors of the mean (SEM).

The overall ANOVA on d’ had the between-subjects variable animation mode (static [Exp. 1] vs. dynamic [Exp. 2]) and repeated measures on learning modality (FV vs. V) and cycle pairs (1_2; 3_4; and 5_6). We observed an interaction between learning modality and cycle pairs (*F*[2,60] = 3.28, *p* = .045, *η*_*p*_^2^ = .098), suggesting costs of bimodal (FV) compared to unimodal learning (V) of voices for the first cycle pair (1_2), and benefits in the third cycle pair (5_6), cf. Fig 2. Polynomial trends analysis indicated that the interaction between learning modality and cycle pairs was due to differences in the linear trend (*F*[1,30] = 6.82, *p* = .014, *η*_*p*_^2^ = .185), without any difference between learning modalities in the quadratic trend (*F*[1,30] < 1). Separate analyses of the FV and V conditions revealed a linear trend in the FV condition, *F*(1, 30) = 13.759, *p* < .001, *η*_*p*_^2^ = .314 with no significant trends in the V condition (*F*[1,30] < 1). We also performed a polynomial trend analysis on d’ across all 6 study-test cycles, despite the fact that the power of this analysis was likely compromised by the small number of trials per condition, with only 6 studied and 6 novel voices per single cycle. This analysis did not reveal a prominent interaction (*F*[1, 30] = 2.735, *p* = .109, *η*_*p*_^2^ = .084), although a linear trend was observed for the FV condition (*F*[1, 30] = 5.726, *p* = .023, *η*_*p*_^2^ = .160) but not for the V condition (*F*[1, 30] < 1) when tested separately.

Although d’ difference scores (FV-V) were not significantly different from zero when assessed for individual cycle pairs and collapsed across animation modes (-1.475 ≤ *t*s[31] ≤ 1.507, .142 ≤ *p*s ≤ .867, uncorrected), there was a significantly larger bimodal learning effect in the third (5_6) compared to the first (1_2) cycle pair, *t*(31) = -2.66, *p* = .036, Bonferroni-corrected for three comparisons (*p* = .012 uncorrected). The remaining two post-hoc comparisons of d’ difference scores (FV-V) between cycle pairs 1_2 vs. 3_4 and 3_4 vs. 5_6 did not reach significance. In line with the previous analysis on d´ scores, a polynomial trend analysis on the bimodal learning effect confirmed that there was an increasing benefit of FV over V learning from the first to the third cycle pair (*F*[1, 31] = 7.083, *p* = .012, *η*_*p*_^2^ = .186). The same analysis performed on 6 study-test cycles did not yield any significant effects. Please see Table 1 for mean d’ in each experimental condition.

**Table 1:** Mean d’ (±SEM) for the factors face animation mode (static vs. dynamic), learning modality (face-voice [FV] vs. voice [V]), and cycle pairs (1_2; 3_4; 5_6).

Face Animation	Learning	Cycle Pairs		
Mode	Modality	1_2	3_4	5_6
Static (Exp. 1)	FV	0.60 (0.15)	1.00 (0.14)	0.83 (0.17)
	V	0.93 (0.17)	0.99 (0.19)	0.75 (0.17)
Dynamic (Exp. 2)	FV	0.65 (0.15)	0.87 (0.14)	1.06 (0.17)
	V	0.81 (0.17)	0.83 (0.19)	0.72 (0.17)
Overall (Exp. 1 & 2)	FV	0.63 (0.10)	0.94 (0.10)	0.95 (0.12)
	V	0.87 (0.12)	0.91 (0.14)	0.73 (0.12)

In terms of criterion (C) participants had a liberal response bias overall with C = -.11. An ANOVA on C with animation mode (static v. dynamic) as between-subjects factor, and with learning modality (FV vs. V) and cycle pairs (1_2; 3_4; and 5_6) as within-subjects factors, revealed only a main effect of cycle pairs (*F*[2,60] = 7.88, *p* = .001, *η*_*p*_^2^ = .208). This effect reflected a decrease of C–in other words, an increasingly liberal response bias–with increasing cycle pairs: pair 1_2 (-.022) < pair 3_4 (-.149) < pair 5_6 (-.159).

**Fig 3:**
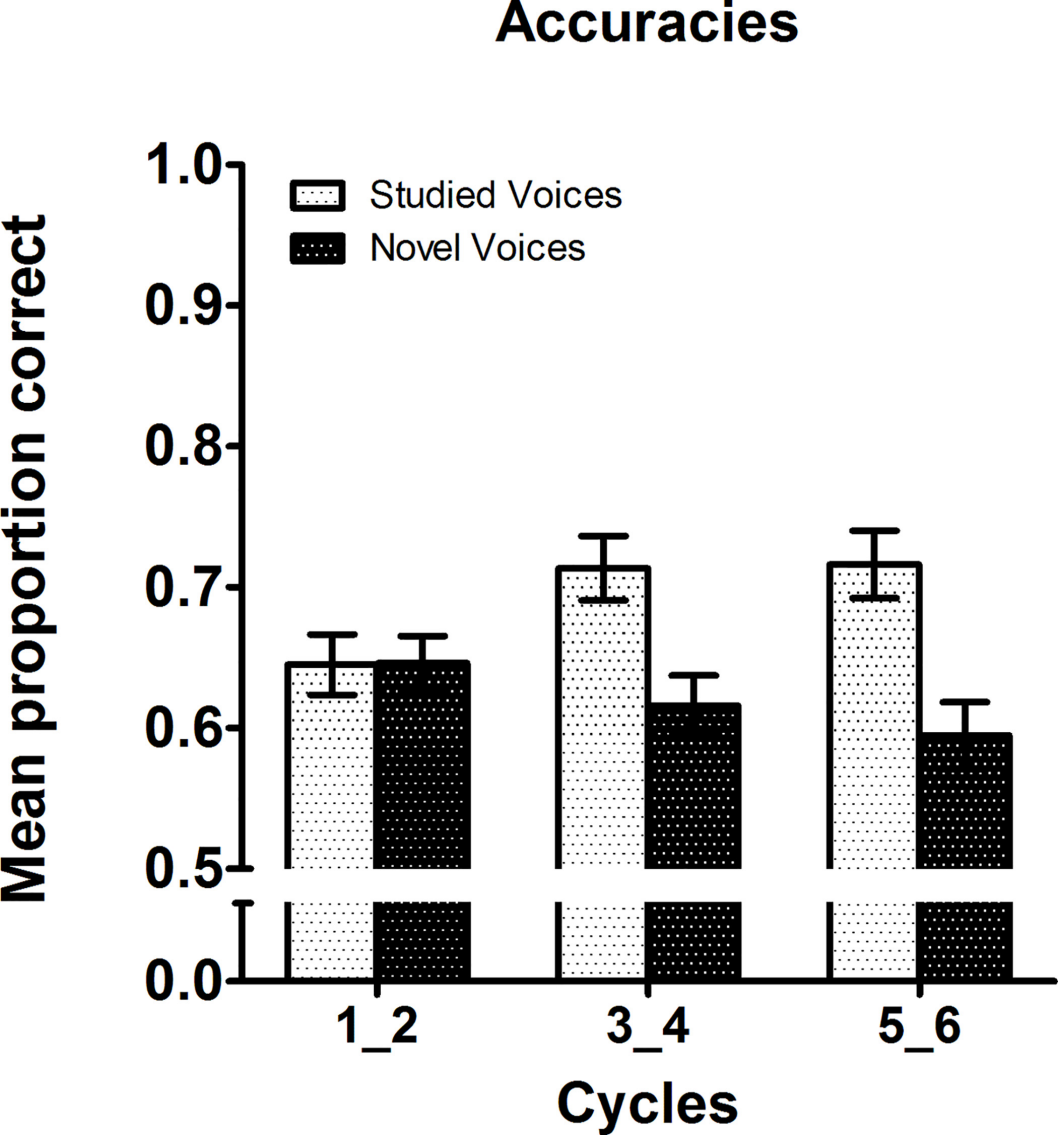
Mean proportion of correct responses to studied vs. novel voices depicted for pairs of consecutive study-test cycles. Note that data are collapsed across learning modality (FV and V) and animation mode (static [Exp. 1] and dynamic [Exp. 2]). Error bars are SEM.

The same ANOVA performed with the additional within-subjects factor voice novelty (studied vs. novel) on accuracies yielded a main effect of novelty (*F*[1,30] = 6.49, *p* = .016, *η*_*p*_^2^ = .178) with more correct responses for studied (hits = .69) versus novel (CR = .62) voices. An interaction of cycle pairs and novelty (F[2,60] = 15.86, *p* < .001, *η*_*p*_^2^ = .346) reflected an increase of hits and a decrease of CR with increasing cycle pairs, cf. Fig 3. These effects were independent of animation mode (static or dynamic) and learning modality (FV or V).

**Fig 4:**
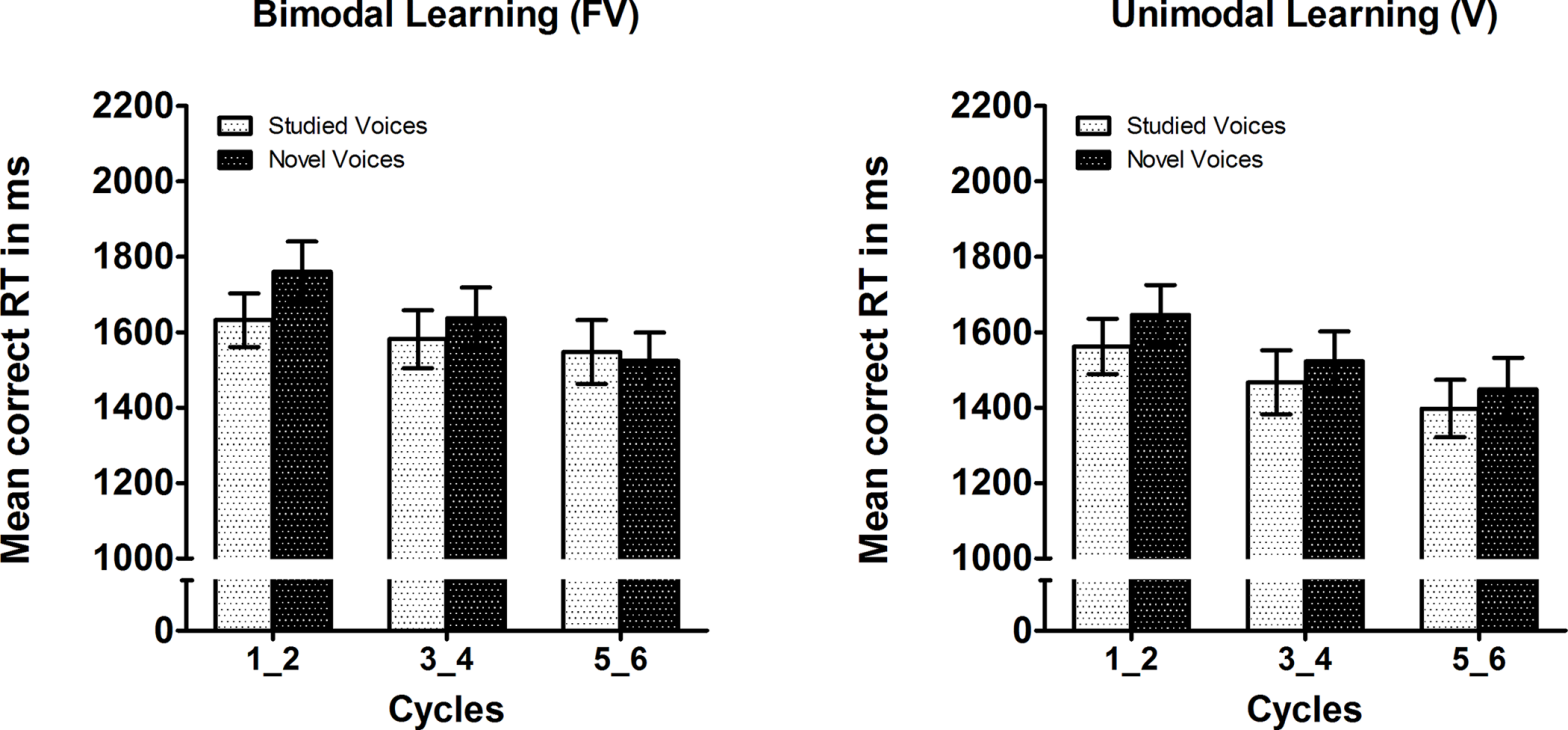
Mean correct reaction times to studied vs. novel voices depicted for pairs of consecutive study-test cycles following (left) bimodal face-voice learning and (right) unimodal voice-only learning. Note that data are collapsed across animation mode (static [Exp. 1] and dynamic [Exp. 2]). Error bars are SEM.

For correct RTs an analogous ANOVA yielded main effects of modality (*F*[1,30] = 4.65, *p* = .039, *η*_*p*_^2^ = .134) with faster responses following unimodal learning compared to bimodal learning, a main effect of cycle pairs (F[2,60] = 15.49, *p* < .001, *η*_*p*_^2^ = .341) reflecting accelerated responses with increasing cycles, as well as a trend for an interaction of voice cycle pairs x novelty (F[2,60] = 3.06, *p* = .054, *η*_*p*_^2^ = .092). This trend pointed to a decrease of the novelty effect with increasing cycle pairs, cf. Fig 4. The above effects were independent of animation mode and learning modality.

## Discussion

Here we demonstrate that intentional learning of unfamiliar voices from two short sentences is increasingly facilitated by the presence of the speakers’ faces, as reflected in sensitivity measures (d-prime). More specifically, while face-voice learning seemed to impair subsequent old/new recognition of voices during the first two (of six) study-test cycles relative to voice-only learning, it gradually improved voice recognition towards the last two study-test-cycles. This pattern of a gradually changing impact of face information on voice recognition is remarkable as previous studies on voice recognition either reported facilitation [7], interference [8, 11, 12] or null effects [9, 10] of face-voice over voice-only learning. In order to reconcile these conflicting results Stevenage et al. [12] recently discussed a number of possible mechanisms. These included context reinstatement between study and test, interference by visual attentional capture (face-overshadowing), additional tasks during encoding, floor effects due to task difficulty, and incidental vs. intentional learning conditions. However, based on the present results we suggest that previous findings may have emerged due to another difference between studies, namely the learning stage they captured. Accordingly, while faces may initially capture attention and thereby cause costs for voice recognition in terms of an overshadowing effect [8, 11, 12], facilitatory effects of audiovisual integration [7] may gradually override face interference as observers acquire more robust audiovisual representations of speakers during learning. Null effects of bimodal learning [9, 10] could therefore reflect an equilibrium between the inhibitory and facilitatory effects of face-overshadowing and audiovisual integration respectively. To fully explain this range of findings, more systematic research on the time course of face-overshadowing and the conditions under which it occurs appears warranted.

Unexpectedly, the present bimodal learning effect was statistically independent of whether faces were shown as static pictures (Exp. 1) or as dynamic videos (Exp. 2), even though Fig 2 suggests a numerical tendency for greater benefits from dynamic than static faces. This null effect is at variance with findings that identity information from dynamic relative to static faces is integrated with vocal cues for personally familiar speakers, but less so for unfamiliar speakers [3]. Specifically, familiarity decisions for personally familiar voices in that study were accelerated by concurrent dynamic faces compared to static face images of the respective speakers. Conversely, identity-incongruent faces presented along with the voices elicited larger response inhibition in reaction times when faces were presented as dynamic videos rather than static images. These results suggest that, at least for highly familiar speakers, there may exist long-term representations of speaker-specific articulatory movements which either match or conflict with the observed movements. Apart from power issues, the present lack of a significant dynamic face advantage during bimodal learning could merely reflect a lower level of familiarity with speakers in the present study compared to personally familiar speakers in Schweinberger’s et al. study. One reason why the present design is hardly suited to create high familiarity with the speakers’ articulation patterns may be the relatively limited variability of speech material in the present stimuli. Thus, compared to static images, videos of speakers uttering two short study sentences may not have provided significant additional information about the speakers’ idiosynchratic articulation patterns. If it is true that voice recognition depends on the phonemic variety of a speech sample (e.g. [18, 19, 20], but see [21]) then bimodal learning may have been compromised by the limited variability of visual speech cues. Note, however, that we observed significant voice learning in all conditions with d’ above chance despite study-to-test-changes of sentence content. This is in line with findings that voice learning leads to the establishment of abstract voice representations that are relatively invariant to speech content [22].

In terms of accuracies there were more correct responses for studied compared to novel voices which converges well with participants’ liberal response bias. In line with these findings, the tendency to find liberal response biases in voice recognition experiments is well known, and stands in contrasts with a tendency for conservative response biases in face recognition experiments (e.g. [23]). These results are also broadly consistent with findings that “familiar only” responses are particularly frequent in voice recognition, when compared with faces [24, 25]. In the present study, the liberal bias intensified over the course of the study-test cycles possibly due to an increased feeling of familiarity also for novel voices. Restrictions to the number of different voices available for the experiment necessitated the repetition of both study voices and novel test voices. This may have led to increasing source uncertainty at test, i.e. uncertainty whether a given voice was familiar from the preceding study phases or for being a “novel” voice from previous test phases. In reaction times this was reflected in a trend for a decrease of an initial novelty effect: faster responses to studied vs. novel voices in the first cycles, but similar response times in the last cycles. A general speeding of responses towards the end of the experiment likely reflects a general training effect. Interestingly, increased reaction times were observed following FV compared to V learning suggesting an overall disadvantage of processing efficiency induced by previous face-voice learning. FV and V learning conditions were presented in separate blocks. It is therefore likely that participants used time-consuming visual imagination strategy for the speakers’ faces during voice retrieval after FV learning. After V learning, by contrast, participants may not have engaged much in visual imagery knowing only voices had been presented during study. This hypothesis could be tesed behaviourally with a design that randomly mixes FV and V learning trials. Alternatively, neuroimaging might reveal whether or not there are activations of face-selective brain areas during voice recognition following FV but not V learning. Since such activations have been previously reported during the recognition of well-known voices [26], such a finding which would be further support for this hypothesis.

Overall, whether or not faces assist voice learning may crucially depend on the salience of both stimuli or the ability to overcome distraction from the face. This may be harder in the case of voice learning with faces than in the reverse situation, as faces seem to be the dominant stimulus domain for speaker recognition [11, 12]. Based on the present data we conclude that two opposing mechanisms take place during bimodal voice learning at different stages of learning: (1) in initial stages of voice learning, attentional capture by faces causes a “face overshadowing effect”, and hence costs to voice learning (2) in later stages of learning, audiovisual integration of redundant identity cues causes a beneficial effect of faces for voice learning.
